# Behavioral and Cognitive Performance Following Exposure to Second-Hand Smoke (SHS) from Tobacco Products Associated with Oxidative-Stress-Induced DNA Damage and Repair and Disruption of the Gut Microbiome

**DOI:** 10.3390/genes14091702

**Published:** 2023-08-27

**Authors:** Jacob Raber, Keaton Stagaman, Kristin D. Kasschau, Conor Davenport, Leilani Lopes, Dennis Nguyen, Eileen Ruth Torres, Thomas J. Sharpton, Glen Kisby

**Affiliations:** 1 Department of Behavioral Neuroscience, 3181 SW Sam Jackson Park Road, Portland, OR 97239, USA; est4003@med.cornell.edu; 2 Departments of Neurology, and Radiation Medicine, Division of Neuroscience, ONPRC, 3181 SW Sam Jackson Park Road, Portland, OR 97239, USA; 3 Department of Microbiology, Oregon State University, Corvallis, OR 97331, USA; kstagman@gmail.com (K.S.); kristin.kasschau@oregonstate.edu (K.D.K.); thomas.sharpton@oregonstate.edu (T.J.S.); 4 Department of Basic Medical Sciences, Western University of Health Sciences, College of Osteopathic Medicine of the Pacific Northwest, Lebanon, OR 97355, USA; conor.davenport@westernu.edu (C.D.); leilani.lopes@westernu.edu (L.L.); duy.nguyen@westernu.edu (D.N.); 5 Department of Statistics, Oregon State University, Corvallis, OR 97331, USA

**Keywords:** second-hand smoke, gut microbiome, cognitive performance, 8-OH-dG, APE1, p21, novel object recognition

## Abstract

Exposure to second-hand Smoke (SHS) remains prevalent. The underlying mechanisms of how SHS affects the brain require elucidation. We tested the hypothesis that SHS inhalation drives changes in the gut microbiome, impacting behavioral and cognitive performance as well as neuropathology in two-month-old wild-type (WT) mice and mice expressing wild-type human tau, a genetic model pertinent to Alzheimer’s disease mice, following chronic SHS exposure (10 months to ~30 mg/m^3^). SHS exposure impacted the composition of the gut microbiome as well as the biodiversity and evenness of the gut microbiome in a sex-dependent fashion. This variation in the composition and biodiversity of the gut microbiome is also associated with several measures of cognitive performance. These results support the hypothesis that the gut microbiome contributes to the effect of SHS exposure on cognition. The percentage of 8-OHdG-labeled cells in the CA1 region of the hippocampus was also associated with performance in the novel object recognition test, consistent with urine and serum levels of 8-OHdG serving as a biomarker of cognitive performance in humans. We also assessed the effects of SHS on the percentage of p21-labeled cells, an early cellular marker of senescence that is upregulated in bronchial cells after exposure to cigarette smoke. Nuclear staining of p21-labeled cells was more prominent in larger cells of the prefrontal cortex and CA1 hippocampal neurons of SHS-exposed mice than in sham-exposed mice, and there was a significantly greater percentage of labelled cells in the prefrontal cortex and CA1 region of the hippocampus of SHS than air-exposed mice, suggesting that exposure to SHS may result in accelerated brain aging through oxidative-stress-induced injury.

## 1. Introduction

Exposure to second-hand smoke (SHS) remains prevalent. While the estimated mortality decreased between 1990 and 2006, it has increased since 2006 [1]. Exposure to SHS is also associated with the development of neurological impairments [2,3,4] and increases the risk of developing neurodegenerative disorders like stroke, mild cognitive impairment (MCI) [2,5,6,7], and dementia [8,9,10], but reduces the risk of developing Parkinson’s disease [11] (for a review, see [12]. The mechanisms underlying the long-term effects of SHS on the brain require elucidation.

The gut microbiome plays an important role in the synthesis of enzymes, amino acids and neurotransmitters, as well as the production of metabolites promoting epithelial barrier integrity, immune modulation, and cognition [13,14]. As a result, the gut microbiome can impact behavioral and cognitive performance through the gut–brain axis, which include vagal nerve innervation, neurotransmitter production, endocrine signaling, and inflammation [15,16,17,18,19,20]. Perturbations to the gut microbiome can result in dysbiosis, wherein the microbiome drives a variety of pathologies that can negatively affect the brain via the gut–brain axis. In humans, the gut microbiome diversifies with age, reflecting healthy aging, and predicts survival [21], while increasing evidence supports the role of an altered gut microbiome in neurodegenerative conditions [22].

Smoking affects the gut microbiome [23], and smoking behaviors may be influenced by specific taxa that produce neurotransmitter-associated metabolites, such as tryptophan and tyrosine [24]. The inhalation of SHS affects both the gut tissue and the composition of the gut microbiome. These observations raise the question of whether environmental exposure to SHS might impact the gut microbiome and thus cause cognitive injury. We hypothesize that SHS inhalation drives changes in the gut microbiome, impacting behavioral and cognitive performance and neuropathology.

In our earlier study [25], we started to assess the effects of chronic SHS exposure (10 months to ~30 mg/m^3^) on behavioral and cognitive performance, metabolism, and neuropathology in 2-month-old wild-type (WT) mice and mice expressing wild-type human tau, a genetic model pertinent to Alzheimer’s disease in which there is a spread of neurofibrillary tangles, consisting of hyperphosphorylated tau aggregates, that is associated with disease severity [26,27]. It is unclear whether SHS induces dysregulation of wild-type human tau. In our current gut microbiome analysis, we included a non-mutant human tau (htau) mouse model that exhibits age-dependent tau dysregulation, neurofibrillary tangles, neuronal loss, neuroinflammation, and oxidative stress starting at 3–4 months and in which tau dysregulation and neuronal loss correlate with synaptic dysfunction and cognitive decline [28].

In addition to behavioral and cognitive performance and neuropathology, the lungs of mice were examined for pathology and alterations in gene expression. We originally hypothesized that WT mice would be less susceptible to the effects of chronic SHS than human tau mice and that WT male mice would be less susceptible to the effects of chronic SHS than WT females. However, our results revealed that the brains of WT mice, and especially WT male mice, were susceptible to the effects of chronic SHS exposure [25]. In a follow-up study, we reported increased levels of 8-hydroxy-2′-deoxyguanosine (8-OH-dG), a marker of oxidative DNA damage and a biomarker of DNA damage following exposure to cancer-causing agents [29], generated following oxidative-stress-induced damage to 2′-deoxyguanosine in the prefrontal cortex of SHS as compared to air controls and a trend towards increased levels in the CA1 area of the hippocampus [30]. In the prefrontal cortex, levels of the oxidative DNA repair marker AP endonuclease 1 (APE1), which is involved in the repair of oxidative DNA damage, were also higher in SHS than air-exposed mice [30]. SHS might also increase various markers of cell senescence in the brain following the oxidative DNA damage, such as β-galactosidase [31,32,33]. 

In the current study, we assessed whether the composition of the gut microbiome from the mice in this prior study varies (1) as a result of SHS exposure and (2) whether the composition of the gut microbiome is linked to behavioral or cognitive phenotypes in these mice. In addition, we assessed whether the oxidative-stress-induced DNA damage that was higher in the hippocampus and prefrontal cortex of SHS-exposed mice correlated with performance in the object recognition test. As β-galactosidase is a marker of senescent cells [34] and cigarette smoke induces p21 expression [35], an early marker of senescence, we also assessed whether β-galactosidase and p21 levels were elevated in the hippocampus or prefrontal cortex following SHS exposure.

## 2. Materials and Methods

### 2.1. SHS Exposure and Behavioral and Cognitive Data

We collected tissues and fecal samples of all 62 mice (*n* = 8 mice/genotype/sex/exposure condition; 1 htau male and 1 wild-type male mouse exposed to SHS died) following open field testing generated as part of a previously NIEHS-funded R21 proposal for the current study. In this project, we assessed the effects of chronic SHS exposure (Scireq inExpose system (Montreal, Quebec, H2S 3G8, Canada; 90% side stream, 10% main stream; 10 months (312 days), 7 days per week, to ~30 mg/m^3^) on behavioral and cognitive performance, metabolism, and neuropathology in 2-month-old wild-type (WT) and human tau mice. For a detailed description of the SHS chemical composition, animal survival, behavioral tests and related results, please see our earlier reported study [25]. To avoid possible withdrawal symptoms in the mice, the mice were behaviorally tested during the last part of the SHS or air exposures. In addition, the lungs of mice were examined for pathology and alterations in gene expression. Details regarding the exposures and analyses reported so far are described in [25]. Briefly, mice were assigned to SHS or air as control exposure and exposed to SHS (90% sidestream and 10% mainstream, using the SCIREQ^®^ inExposeTM system) or air for 168 min per day. Each exposure day, a cigarette-smoking robot (CSR) and a CSR lighter from SCIREQ^®^ were used to light twenty-four 3R4F certified cigarettes (University of Kentucky, Lexington, KY, USA). There was one puff per minute; the flow rate was 2 L/min. Gravimetric analysis was used to analyze the particulate matter count monthly. All procedures were performed according to the National Institutes of Health (NIH) Guide for the Care and Use of Laboratory Animals following approval from the Oregon Health & Science University (OHSU) Institutional Animal Care and Use Committee (IACUC) and consistent with the Animal Research: Reporting of In Vivo Experiments (ARRIVE) guidelines.

### 2.2. 16S Gut Microbiome Analysis

The 16S rRNA gene sequence data were generated from stool collected from mice following our prior work [36]. Briefly, we followed the Earth Microbiome Project protocols to extract DNA using the QIAamp PowerFecal Pro DNA extraction kits and amplify the V4 hypervariable locus using Polymerase Chain Reaction (PCR). Cleaned amplicons were pooled at equimolar concentrations and subject to DNA sequencing on an Illumina MiSeq via the Center for Quantitative Life Sciences at Oregon State University. Sequence data were then demultiplexed, adapter trimmed, and subject to Amplicon Sequence Variant (ASV) clustering using the DADA2 workflow in the R programming environment. 

We evaluated how the α-diversity and β-diversity of the microbiome associates with various experimental covariates, including cohort, sex, and genotype, as well as 10 behavioral physiological covariates. α-diversity is a measure of the biodiversity of the microbiome (i.e., how many different taxa reside in the community). This measurement is agnostic to the specific types of bacteria that comprise the microbiome and instead summarizes ecological characteristics of the community, such as the carrying capacity. β-diversity, on the other hand, is a measure of the composition of the microbiome. In particular, it assesses which specific taxa reside in a community and how the taxonomic composition of the community differs from that of other communities. In effect, β-diversity allows us to assess whether the types of taxa that comprise a microbiome vary as a function of different experimental parameters (e.g., exposure to SHS). For both α- and β-diversity, we evaluated a variety of metrics that similarly summarize these properties of the microbiome, but that differ in their specific mathematical form, which allows us to evaluate different aspects of the ecology of the communities. 

Our analysis of the gut microbiome data followed our prior work [36]. Briefly, we used robust hypothesis tests or linear regression to associate the α-diversity with study covariates, including genotype, exposure to SHS, and sex, using a step-wise model construction framework that zeroes-in on the set of covariates, as well as their potential interaction, that significantly explain the variation in α-diversity across samples (*p* < 0.05). We used conditional correspondence analysis via a related step-wise multivariate regression approach to link covariates or their statistical interaction to β-diversity (*p* < 0.05).

### 2.3. Immunohistochemistry

As the most profound effects of SHS in our earlier published study were seen in wild-type male mice, we only used wild-type male mice for the immunohistochemical analyses (*n* = 3 mice/exposure condition).

*Antibodies.* The immunohistochemistry was carried out using a mouse monoclonal antibody (15A3) raised against 8-hydroxyguanosine (8-OHdG) from Santa Cruz Biotechnology (Dallas, TX, USA), anti-APE1 polyclonal antibodies from ThermoFisher and an antibody to the senescence marker p21 from Abcam (ab107099) as described in [25,30]. The quantification of the results of the immunohistochemical analyses for oxidative-stress-induced markers in the CA1 region of the hippocampus and prefrontal cortex are described in Table 1 [30].

The brains (right hemispheres) of the male wild-type mice were submerged in 4% buffered paraformaldehyde, cryoprotected in sucrose (30%) and frozen with Tissue-Tek^®^. Brains from male wild-type mice were selected as this was the most affected group for earlier reported outcome measures in mice chronically exposed to SHS. Cryopreserved brain tissue sections (20 μm) were placed on Superfrost^®^ Plus (VWR/Avantor) (Radnor, PA, USA) glass slides (2 sections/per slide). The slides were airdried and, the next day, processed for staining or immunohistochemistry. The slides were warmed to room temperature and the sections were outlined using an ImmEdge^®^ PAP Pen (Vector Labs, Newark, CA, USA) before staining or immunohistochemistry. Brain tissue sections were subjected to antigen retrieval for 45 min using a heated citrate-based unmasking solution (H-3301, Vector Labs, Newark, CA, USA) and subsequently blocked with Tris-buffered saline with 0.1% Tween^®^ (TBST) (Millipore Sigma, Burlington, MA, USA) for 15 min. The unmasked sections were incubated overnight at 4 °C with the primary antibodies described above and subjected to avidin-biotin peroxidase staining by quenching them for 30 min, before adding a biotinylated secondary antibody (Vectastain^®^ Elite ABC Kit, Newark, CA, USA) and blocking with Bloxall (Vectastain^®^ Elite ABC Kit, Newark, CA, USA) for 10 min. Slides were incubated with Vectastain^®^ Elite ABC reagent and NovaRed^®^ peroxidase substrate (red/brown staining) (Vector Labs, Newark, CA, USA). The slides were washed with deionized/distilled water, dehydrated with alcohol and xylene substitute, mounted with Cytoseal60^®^ (Fisher Scientific, Norwalk, CA, USA) and imaged using a Leica DM100 microscope (Deerfield, IL, USA) at various magnifications.

Three representative images from the prefrontal cortex and hippocampal areas (CA1, CA3, CA4) were manually counted in a blinded fashion, based on the staining intensity compared to the background signal.

### 2.4. Histochemical Staining

Senescence-associated-β-galactosidase (SA-β-gal) staining was performed on brain tissue sections (as described above) using a Cellular Senescence Assay Kit (CBA-230; Cell Biolabs Inc., San Diego, CA, USA) according to the manufacturer’s protocol with minor modifications [37]. Cryopreserved brain tissue sections from both air- and SHS-exposed male wild-type male mice were incubated for 8 h at 37 °C (in the dark) with cell staining solution, the staining solution removed and the sections washed with PBS. The stained sections were imaged on a Leica DM100 microscope (Deerfield, IL, USA) at various magnifications. Three representative images from the prefrontal cortex of air- and SHS-exposed mice were manually counted, the counts averaged and the data analyzed using R as previously described [30].

### 2.5. Statistical Analyses

The statistical analyses for the 16S gut microbiome analysis are described above. For statistical analyses for the staining and immunohistochemistry, the counts from each brain region and exposure were averaged and the data were analyzed using a Welch *t*-test with R (v 4.2.3). Figure representing quantification of the corresponding data (expressed as means ± SD) was generated using GraphPad Prism (v 9.5.1) software. Correlations between the percentage of 8-OH-dG-labeled cells in the CA1 region of the hippocampus and APE1-labeled cells in the prefrontal cortex with performance in the object recognition test were analyzed using Pearson correlations and GraphPad Prism software (v.8).

## 3. Results

### 3.1. α Diversity of the Gut Microbiome Correlates with Cognitive Performance

**Figure 1:**
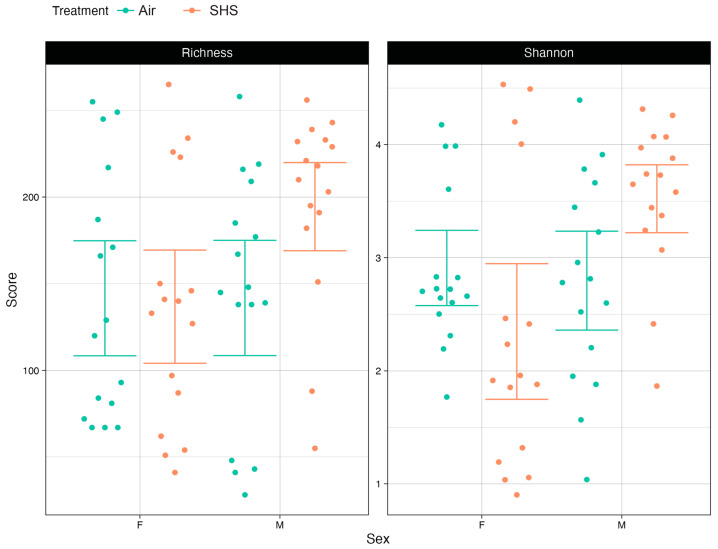
Microbiome richness (i.e., number of taxa in a sample) (**Left**) and Shannon entropy (i.e., number of taxa in a sample weighted by taxon abundance) (**Right**) were increased in male mice exposed to SHS. In contrast, SHS had no detectable effect on measures of α-diversity in female mice. Points represent an α-diversity measure from a single mouse in the study. Horizontal lines represent 95% confident intervals based on a bootstrapping analysis. Samples from SHS-exposed mice are in orange, while samples from air controls are in green. The results are shown with genotype collapsed.

The microbiome samples we interrogated were comprised of taxa typically observed in mouse gut microbiome studies [38,39]. For example, the top ten most abundant genera based on the median relative across all samples were *Faecalibaculum* (28.4%), *Lactobacillus* (12.4%), *Turicibacter* (6.15%), *Alistipes* (3.31%), a genus within the Lachnospiraceae_NKA136_group (2.49%), *Lachnoclostridium* (1.69%), *Rosburia* (1.45%), a genus within the Lachnospiraceae_FC020_group (0.96%), *Muribaculum* (0.96%), and *Desulfovibrio* (0.52%). For all analyses, the microbiome α- and β-diversity did not vary as a function of mouse genotype, indicating that the associations presented here are agnostic to the mouse htau genotype. SHS did not significantly affect the α-diversity of the gut microbiome in a general sense, but it did affect the α-diversity of the gut microbiome in a sex-dependent manner. Specifically, for mice in the air exposure group, differences in α-diversity between female and male mice were negligible. However, for male mice exposed to SHS, the α-diversity of their microbiomes was significantly higher than those of female mice (Figure 1). The diversity of the female mouse microbiomes were comparable to the diversity of the air-exposed mouse microbiomes. We observed this pattern for both measures of α-diversity we considered (Figure 1): richness, which measures the number of distinct microbes present in a community, as well as Shannon entropy, which additionally reflects the evenness of the community in terms of the relative abundance of the various taxa that are present.

**Figure 2:**
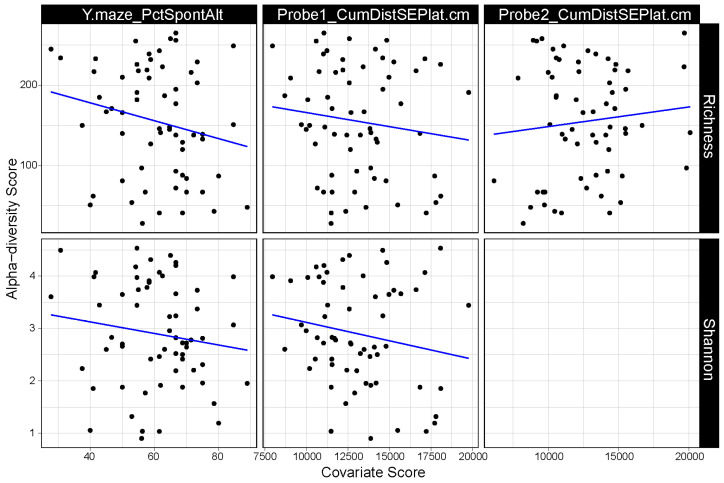
Scatterplots illustrating the relationship between measures of mouse microbiome α-diversity as well as measures of cognitive performance. The top row of plots illustrates results based on microbiome richness (i.e., number of taxa in a sample), while the bottom row illustrates results based on Shannon entropy (i.e., number of taxa weighted by taxon abundance). Each column represents one or more plots based on a particular measure of cognition (Y.maze_PctSponAlt: percentage of spontaneous alterations in a Y maze; Probe1_CumDistSEPlat.cm: cumulative distance to the target location in the first water maze probe trial; Probe2_CumDistSEPlat.cm: cumulative distance to the target location in the second water maze probe trial). Points represent individual microbiome samples. Blue lines illustrate the line of best fit as determined by linear regression. Only significant associations after multiple test correction are shown here.

The α-diversity of the gut microbiome is also associated with several measures of cognitive performance (Figure 2). In the water maze test, hippocampus-dependent spatial retention is assessed in a probe trial (no platform present). Microbiome richness negatively was linked to the cumulative distance to the target location in the first water maze probe trial (*p* = 0.019), while it was positively linked to the same cognitive measure in the second water maze probe trial (*p* = 0.006). Richness was also negatively linked to the percentage of hippocampus-dependent spontaneous alternations in the Y maze (*p* = 0.048).

Measures of Shannon entropy were negatively correlated with the cumulative distance to the target location in the first water maze probe trial (*p* = 0.031) as well as the percentage of spontaneous alternations in the Y maze (*p* = 0.033) (Figure 2).

### 3.2. β-Diversity of the Gut Microbiome Correlates with Cognitive Performance

**Figure 3:**
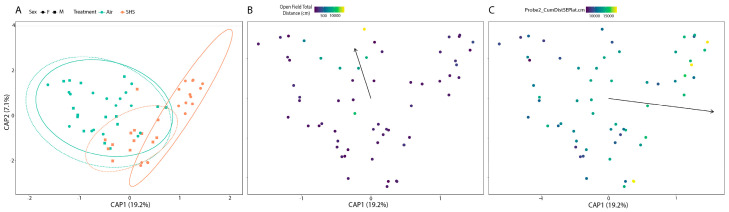
Ordinations of microbiome β-diversity. The Bray–Curtis dissimilarity metric quantified the dissimilarity in community composition across samples while weighting these differences based on the abundance of taxa. These Bray–Curtis dissimilarities were used to generate the distance based redundancy analysis ordination illustrated here, wherein samples are represented by points in the ordination. This ordination is color coded in three forms representing three different analyses: (**A**) the relationship between β-diversity and SHS exposure as well as mouse sex; (**B**) the relationship between β-diversity and distance moved in the open field test; (**C**) the relationship between β-diversity and cumulative distance to the target location in the second water maze probe trial. The main text provides specific information on how to interpret each of the above plots.

The β-diversity analysis revealed that the composition of the gut microbiome–the specific assemblage of microbial taxa that reside in the gut varies as a function of exposure to SHS (*p* = 0.001). However, the composition of male and female mouse microbiomes responded differently to SHS exposure (*p* = 0.002). In addition to multivariate regression, ordinations were used to visualize the similarity in the composition of microbiome samples. In ordinations, each microbiome sample is illustrated as a single point in a coordinate space, wherein the distance between two points within this space represents the extent of difference in the composition of the two microbiome samples these points represent. Points were color coded to illustrate how samples from mice exposed to SHS differ in their composition relative to samples collected from air-exposed mice (Figure 3). Data points were colored based on the exposure group the corresponding sample was collected from. Moreover, the shape of a point represents whether the corresponding sample was collected from a male or female mouse. To aid in interpretation of the data, 95% confidence intervals are placed around each group of points based on the corresponding sample’s exposure (Air versus SHS) and sex (Male versus Female) characteristics. This analysis shows that while male and female mouse microbiomes appear to be statistically indistinguishable for air-exposed mice, male and female mouse microbiomes take on distinct compositions relative to one another in SHS-exposed mice.

The composition of the gut microbiome is also associated with behavioral and cognitive measures: the total distance moved in an open field (*p* = 0.022), a measure of activity and response to novelty, and the cumulative distance to the target location in the second water maze probe trial (*p* = 0.041), a cognitive measure. The results of this analysis are visually illustrated in Figure 3B,C. For these ordinations, points are colored based upon the performance score that the corresponding mouse received in each of these two tests. Higher tests scores are illustrated with brighter colors. A regression analysis evaluated how these test scores were distributed within the ordination space to clarify the relationship between the β-diversity and test performance. In Figure 3B,C, an arrow overlaid onto this ordination space illustrated the results of this regression. For example, mice with a higher cumulative distance to the target location (worse cognitive performance) in the second water maze probe trial tend to be oriented on the right hand side of the ordination space, whereas the mice with lower scores for this test (better cognitive performance) tend to be oriented on the left hand side of this ordination space.

### 3.3. β-Galactosidase

**Figure 4:**
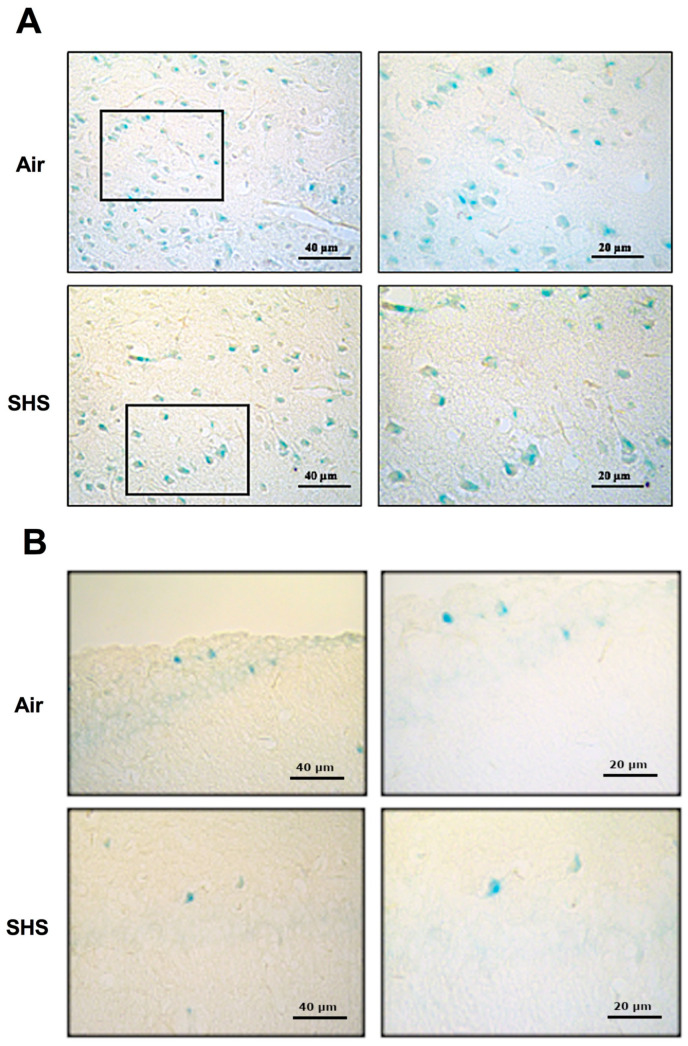
Senescence-associated β-galactosidase (SA-β-gal) staining in the prefrontal cortex (**A**) and the hippocampus (**B**) of mice chronically exposed to SHS. Higher magnifications of the prefrontal cortex (*boxes:* 34.7x). Stained sections from air- and SHS-exposed mice (*n* = 3/tx) were manually counted for β-Gal staining, as previously described [30]. There was no effect of SHS on β-Gal staining in either brain region.

Increased activity of β-galactosidase (β-gal) is a common biomarker of senescent cells in the brain [40]. We assessed the effects of SHS on the percentage of senescence-associated biomarker β-galactosidase (SA-β-gal)-labeled cells in wild-type male mice. There was no effect of SHS on the percentage of SA-β-gal-labeled cells in the CA1 region of the prefrontal cortex (Figure 4A) or hippocampal CA1 region (Figure 4B). SA-gal staining was also similar across other regions of the hippocampus.

### 3.4. Relationship between Percentages of p21-Labeled Cells in the CA1 Region of the Hippocampus and the Prefrontal Cortex

**Figure 5:**
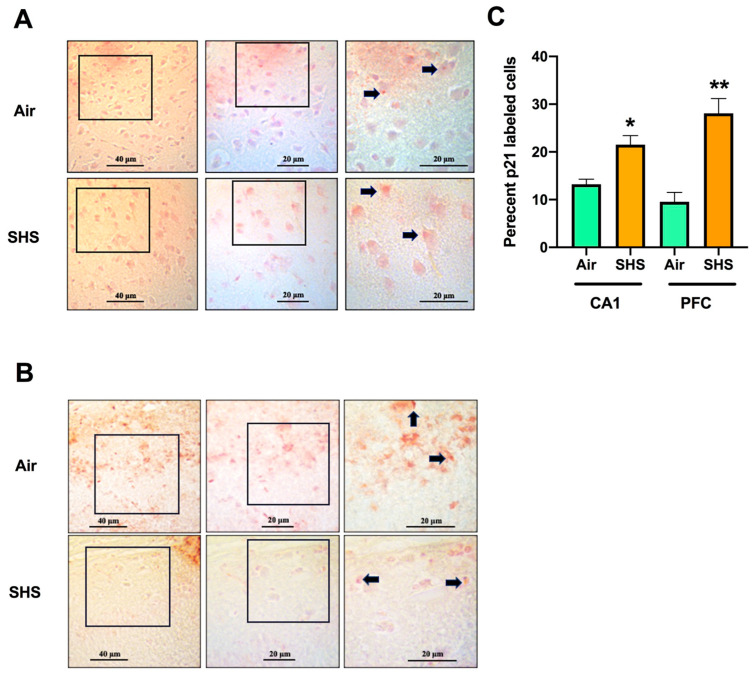
p21 staining in the prefrontal cortex (**A**) and CA1 region of the hippocampus (**B**) of mice chronically exposed to SHS. These brain areas were stained for the senescence marker p21. Note that there was prominent staining of cells within the prefrontal cortex and the hippocampal CA1 region of SHS- than air-exposed mice (“*arrows*”). Higher magnifications (boxes) of the prefrontal cortex (middle and right images in (**A**) indicate that the nuclear staining was more prominent in larger cells. (**C**) Stained sections from both air- and SHS-exposed mice were manually counted for p21 staining, as previously described [42]. The percentages of labelled cells in the prefrontal cortex and hippocampal CA region of SHS-exposed mice were significantly greater than air-exposed mice. Values are expressed as percentages of labeled cells. * *p* < 0.05, ** *p* < 0.01, 2-tailed *t*-tests.

We also assessed the effects of SHS on the percentage of p21-labeled cells, an early cellular marker of senescence [41] that is upregulated in bronchial cells after exposure to cigarette smoke [42]. Nuclear staining of p21-labeled cells was more prominent in larger cells of the prefrontal cortex (Figure 5A) and CA1 hippocampal neurons (Figure 5B) of SHS-exposed mice than in sham-exposed mice. This was consistent with a significantly greater percentage of labelled cells in both the prefrontal cortex and hippocampus (CA1 neurons) of SHS- than air-exposed mice (*p* = 0.007 and *p* = 0.0191, respectively) (Figure 5C). In contrast, p21 staining was not different in other regions of the hippocampus.

### 3.5. Relationship between Percentages of 8-OH-dG-Labeled Cells in the CA1 Region of the Hippocampus and APE1-Labeled Cells in the Prefrontal Cortex with Performance in the Object Recognition Test

**Figure 6:**
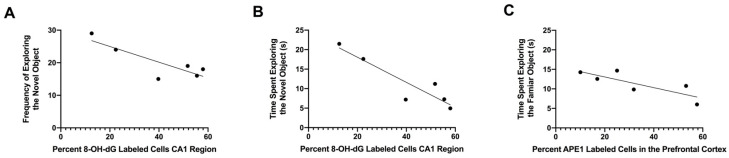
(**A**) The percentage of 8-OH-dG-labeled cells in the CA1 region of the hippocampus was negatively correlated with the frequency of exploring the novel object (r = −0.8482, *p* = 0.0326, Pearson. (**B**) The percentage of 8-OH-dG-labeled cells in the CA1 region of the hippocampus was negatively correlated with the time spent exploring the novel object (r = −0.9182, *p* = 0.0098, Pearson). (**C**) The percentage of APE1-labeled cells in the CA1 region of the hippocampus was negatively correlated with the time spent exploring the familiar object (r = −0.8176, *p* = 0.0469, Pearson).

We also examined the effect of oxidative-stress-induced DNA damage and repair (Table 1) on both behavioral performance and cognition in SHS-exposed mice. We assessed the relationships between these biomarkers of SHS exposure and performance in the object recognition test. The percentage of 8-OH-dG-labeled cells in the CA1 region of the hippocampus was negatively correlated with the frequency of exploring the novel object (r = −0.8482, *p* = 0.0326, Pearson (Figure 6A)) and the time spent exploring the novel object (r = −0.9182, *p* = 0.0098, Pearson (Figure 6B) in the object recognition test. In addition, the percentage of APE1-labeled cells in the prefrontal cortex was negatively correlated with the time spent exploring the familiar object (r = −0.8176, *p* = 0.0469, Pearson (Figure 6C)).

There was no correlation between the percentage of β-gal-labeled cells in the prefrontal cortex and time or frequency exploring the novel object in the object recognition test. The quantification of the results of the immunohistochemical analyses for oxidative-stress-induced markers in the CA1 region of the hippocampus and prefrontal cortex is summarized in Table 1.

## 4. Discussion

The results of this study indicate that SHS exposure significantly impacts the composition of the gut microbiome, and that these changes are linked to cognitive impairments. The α-diversity analyses revealed that SHS impacts the biodiversity and evenness of the gut microbiome in a sex-dependent fashion and that these same measures of the gut microbiome broadly link to cognitive performance. Consistent with our earlier cognitive, lung and brain pathological data and plasma and brain metabolomics data [25], chronic SHS exposure also had a significant impact on the gut microbiome of male mice as indicated by the richness, evenness, and composition of the gut microbiome that associated with several measures of cognitive performance. These observations are consistent with prior studies that link SHS exposure to changes in the microbiome, as well as prior studies—including our own—that link mouse cognitive performance and the gut microbiome. However, our analysis is the first to consider how chronic SHS exposure impacts the gut microbiome, and it is also the first to intertwine measures of cognition into our understanding of the impacts of SHS on the microbiome. Collectively, these results support the hypothesis that the gut microbiome contributes to the effect of SHS exposure on cognition. Additional studies will be needed to verify the causal role of the microbiome in this process.

The water maze and Y maze assess hippocampus-dependent learning and memory, so the results of this study generally point to a connection between the α-diversity of the gut microbiome and hippocampus-dependent learning and memory. That said, the opposing associations between richness and the two subsequent water maze probe trial test results indicate that the observed relationship between α-diversity and spatial memory retention may be complex; while the first water maze trial occurred 24 h after the mice were trained for two days to locate a hidden platform, the second water maze trial took place 72 h after the mice were trained for three days to locate the hidden platform. We postulate that the different association between performance in these two probe trials and the gut microbiome relates to the different challenges associated with these two spatial memory retention tests due to the distinct intervals between the last hidden platform training trial and the spatial memory assessment.

We recognize that because of the environmental challenge in the SHS study, 10 months of SHS exposure for 7 days per week, there could be an effect of aging as well. In light of the long SHS exposure, the genotype difference in effects of SHS on the gut microbiome might be subtle. This is of translational relevance considering environmental exposure to SHS in humans with different genetic backgrounds. What is striking is that there is a profound sex difference that is apparently stronger than a possible htau genotype effect. This is an important result because our prior gut microbiome studies also found that genotype and sex effects on the gut microbiome significantly correlated with behavioral changes [38,39,43]. The percentage of 8-OH-dG-labeled cells in the CA1 region of the hippocampus, involving the formation, consolidation, and retrieval of hippocampal-dependent memories and the main output of the hippocampus [44], also correlated negatively with the frequency of exploring the novel object and the time spent exploring the novel object in the object recognition test. Consistent with these data, elevated urinary 8-OHdG levels, a biomarker of smoking status [45], were associated with lower global cognitive scores in 45–75-year-old adults, after adjustment for age, education, and the genetic risk factors for age-related cognitive decline and Alzheimer’s disease apolipoprotein E4 [46]. Urinary 8-OHdG levels were used as a biomarker to distinguish Alzheimer’s patients from cognitively healthy controls [47,48]. Similarly, higher serum levels of 8-OH-dG 24 h after hospital admission were associated with early cognitive impairments, as assessed by the Mini-Mental State Exam 30 days later, in patients with stroke [49]. However, this biomarker is not specific for cognitive injury as higher serum levels of 8-OH-dG 24 h after hospital admission were also associated with depression, as assessed by the Hamilton Depression Score, 30 days later [50]. These data are consistent with the hypotheses that peculiar microbiota might induce oxidative stress in the brain [51,52] and, vice versa, that brain injury might affect the gut microbiome [53].

Oxidative DNA damage is repaired by both the base-excision pathway and APE1-initiated activation of the non-homologous end joining pathway in cortical neurons [54,55]. The percentage of APE1-labeled cells in the prefrontal cortex also correlated negatively with the time spent exploring the familiar object. Unless exploring the familiar object less is associated with exploring the novel object more, this is not a cognitive measure. These data suggest that APE1 might be more a general marker of oxidative-stress-induced DNA damage and senescence, but not of cognitive performance.

Oxidative-stress-induced DNA damage can also induce changes in the neuronal cell cycle to activate a persistent DNA damage response, leading to neuronal senescence [56]. SHS exposure increased the levels of p21-labeled cells in the prefrontal cortex and hippocampus, but not β-galactosidase activity, a widely used marker of cellular senescence that is also elevated in neurons with high energetic and metabolic demands [57]. As p21 is an early marker of cell senescence [58], these results suggest that exposure to SHS might result in accelerated brain aging through oxidative-stress-induced injury.

While a unique strength of the experimental design used is that it involves an environmentally controlled exposure pertinent to humans, we recognize the following limitations: (1) as mice were exposed for 10 months, we cannot distinguish chronic effects of SHS from the effect of aging and interactions between the two. Future studies, starting the exposure either earlier or later in life, might help address this concern. (2) While used as a mouse model pertinent to Alzheimer’s disease, the human tau mice express wild-type tau. Future efforts are warranted that include mouse models expressing human mutations of genes that modulate risk to develop neurodegenerative disease. (3) In our paradigm, mice were exposed 168 min per day. It is conceivable that the exposure of mice for longer periods of time per day might cause more pronounced effects on the gut microbiome and brain. Finally, (4) while the current study revealed associations between the gut microbiome and behavioral measures, this does not prove causality. Future studies involving fecal transplants of mice exposed to SHS into germ-free mice should be considered to determine if alterations in the gut microbiome are sufficient to induce phenotypes in the recipient mice.

## 5. Conclusions

In summary, SHS exposure impacts the composition of the gut microbiome, and this in turn is linked to cognitive impairments. The increased levels of 8-OHdG in the CA1 region of the hippocampus were also associated with performance in the novel object recognition test, consistent with urinary and serum levels of 8-OHdG as a biomarker of cognitive performance in humans. SHS exposure also increased APE1 in the prefrontal cortex. Nuclear staining of p21-labeled cells was more prominent in larger cells of the prefrontal cortex and CA1 hippocampal neurons of SHS-exposed mice than in sham-exposed mice, and there was a significantly greater percentage of labelled cells in the prefrontal cortex and CA1 region of the hippocampus of SHS- than air-exposed mice, suggesting that exposure to SHS might result in accelerated brain aging through oxidative-stress-induced injury.

## Figures and Tables

**Table 1:** Summary of immunohistochemical analyses reported in [27] ^1^. Oxidative DNA damage and repair biomarkers in the air- and SHS-exposed mouse prefrontal cortex (PFC) and various regions of the hippocampus (HIPP).

Marker	Region and Description (SHS vs. Air)	% Labelled Cells Air; SHS
**8-OHdG**	*PFC* Darker and more punctate staining throughout	32.7 ± 11.3; 61.5 ± 5.7 *
	*HIPP-CA1* Darker and more punctate staining throughout	30.2 ± 15.8; 55.1 ± 3.1
	*HIPP-CA3* Darker and more punctate staining throughout	29.2 ± 3.5; 57.1 ± 6.3
**OGG1**	*HIPP-CA4* Darker and more punctate staining throughout	40.8 ± 10.1; 58.9 ± 2.9
	*PFC* Darker and more punctate staining in air exposed	**-**
**APE1**	*HIPP* Darker staining of CA1 neurons. Less dark staining in CA2 and CA3 neurons.	**-**
	*PFC* Darker and more punctate staining throughout	17.4 ± 7.5; 47.6 ± 13.8 *
	*HIPP-CA1* Darker and more punctate staining throughout	30.2 ± 15.8; 56.3 ± 4.5
	*HIPP-CA3* Darker and more punctate staining throughout	30.4 ± 9.1; 58.9 ± 8.7 *
	*HIPP-CA4* Darker and more punctate staining throughout	32.7 ± 10.1; 66.3 ± 7.8 *
		**-**

^1^ Brain tissue sections (20 µm) from air- and SHS-exposed mice (*n* = 3 mice/exposure group) were immunohistochemically analyzed for oxidative DNA damage (8-hydroxydeoxyguanosine, 8-OHdG) or base excision repair proteins (apurinic/apyrimidinic endonuclease, APE1; 8-oxoguanine glycosylase, OGG1) as described in Lopes et al. (2023). Significant SHS effects are indicated in bold. * *p* < 0.05, Welch *t*-test with R (v. 4.2.3). PFC: prefrontal cortex; HIPP: hippocampus.

## Data Availability

The16S gut microbiome data set used for this study can be found on the NCBI website.

## References

[1] Yousuf H., Hofstra M., Tijssen J., Leenen B., Lindemans J.W., van Rossum A., Narula J., Hofstra L. (2020). Estimated worldwide mortality attributed to secondhand tobacco smoke exposure. JAMA Netw. Open.

[2] Akhtar W.Z., Andresen E.M., Cannell M.B., Xu X. (2013). Association of blood cotinine level with cognitive and physical performance in non-smoking older adults. Environ. Res..

[3] Heffernan T.M., O’Neill T.S. (2013). Everyday prospective memory and executive function deficits associated with exposure to second-hand smoke. J. Addict..

[4] Heffernan T.M., O’Neill T.S. (2013). Exposure to second-hand smoke damages everyday prospective memory. Addiction.

[5] Orsitto G., Turi V., Venezia A., Fulvio F., Manca C. (2012). Relation of secondhand smoking to mild cognitive impairment in older inpatients. Sci. World J..

[6] Barnes D.E., Haight T.J., Mehta K.M., Carlson M.C., Kuller L.H., Tager I.B. (2010). Tager Secondhand smoke, vascular disease, and dementia incidence: Findings from the cardiovascular health cognition study. Am. J. Epidemiol..

[7] Llewellyn D.J., Lang I.A., Langa K.M., Naughton F., Matthews F.E. (2009). Matthews Exposure to secondhand smoke and cognitive impairment in non-smokers: National cross sectional study with cotinine measurement. BMJ.

[8] Cataldo J.K., Prochaska J.J., Glantz S.A. (2010). Cigarette smoking is a risk factor for Alzheimer’s Disease: An analysis controlling for tobacco industry affiliation. J. Alzheimer’s Dis..

[9] Chen R. (2012). Association of environmental tobacco smoke with dementia and Alzheimer’s disease among never smokers. Alzheimer’s Dement..

[10] Schick S., Glantz S. (2005). Philip Morris toxicological experiments with fresh sidestream smoke: More toxic than mainstream smoke. Tob. Control..

[11] Mappin-Kasirer B., Pan H., Lewington S., Kizza J., Gray R., Clarke R., Peto R. (2020). Tobacco smoking and the risk of Parkinson disease. Neurology.

[12] Liu W., Wang B., Xiao Y., Wang D., Chen W. (2021). Secondhand smoking and neurological disease: A meta-analysis of cohort studies. Rev. Environ. Health.

[13] Davidson G.L., Cooke A.C., Johnson C.N., Quinn J.L. (2018). The gut microbiome as a driver of individual variation in cognition and functional behaviour. Philos. Trans. R. Soc. B Biol. Sci..

[14] Gareau M. (2016). Cognitive function and the microbiome. Int. Rev. Neurobiol..

[15] Foster J.A., McVey Neufeld K.-A. (2013). Gut-brain axis: How the microbiome influences anxiety and depression. Trends Neurosci..

[16] Allen A.P., Dinan T.G., Clarke G., Cryan J.F. (2017). A psychology of the human brain-gut-microbiome axis. Soc. Personal. Psychol. Compass.

[17] Kelly J.R., Kennedy P.J., Cryan J.F., Dinan T.G., Clarke G., Hyland N.P. (2015). Breaking down the barriers: The gut miocrobiome, intestinal permeability and stress-related psychiatric disorders. Front. Cell Neurosci..

[18] Lynch J., Hsiao E. (2019). Microbiomes as sources of emergent host phenotypes. Science.

[19] Vazquez-Villasenor I., Garwood C.J., Simpson J.E., Heath P.R., Mortiboys H., Wharton S.B. (2017). The microbbiome and host behavior. Annu. Rev. Neurosci..

[20] Sudo N., Chida Y., Aiba Y., Sonoda J., Oyama N., Yu X.-N., Kubo C., Koga Y. (2004). Postnatal microbbila colonization programs the hypothalamic-pituitary-adrenal system for stress response in mice. J. Physiol..

[21] Wilmanski T., Diener C., Rappaport N., Patwardhan S., Wiedrick J., Lapidus J., Earls J.C., Zimmer A., Glusman G., Robinson M. (2021). Gut microbiome pattern reflects healthy ageing and predicts survival in humans. Nat. Metab..

[22] Ning J., Huang S.Y., Chen S.D., Zhang Y.R., Huang Y.Y., Yu J.T. (2023). Investigating Casual Associations Among Gut Microbiota, Metabolites, and Neurodegenerative Diseases: A Mendelian Randomization Study. J. Alzheimer’s Dis..

[23] Huang C., Shi G. (2019). Smoking and microbiome in oral, airway, gut and some systemic diseases. J. Trans. Med..

[24] Fan J., Zhou Y., Meng R., Tang J., Zhu J., Aldrich M.C., Cox N.J., Zhu Y., Li Y., Zhou D. (2023). Cross-talks between gut microbiota and tobacco smoking: A two-sample Mendelian randomization study. BMC Med..

[25] Raber J., Perez R., Torres E., Krenik D., Boutros S., Patel E., Chlebowski A., Torres E.R., Perveen Z., Penn A. (2021). Effects of chronic second-hand smoke (SHS) exposure on cognitive performance and metabolic pathways in the hippocampus of wild-type and human tau mice. Environ. Health Perspect..

[26] Dujardin S., Commins C., Lathuiliere A., Beerepoot P., Fernandes A.R., Kamath T.V., De Los Santos M.B., Klickstein N., Corjuc D.L., Corjuc B.T. (2020). Tau molecular diversity contributes to clinical heterogeneity in Alzheimer’s disease. Nat. Med..

[27] Mufson E., Ward S., Binder L. (2014). Prefribillar tau oligomers in mild cogntive impairments and Alzheimer’sdisease. Neuro-Degener. Dis..

[28] Polydoro M., Acker C., Duff K., Casitillo P., Davies P. (2010). Age-dependent impairment of cognitive and synaptic function in the htau mouse model of tau pathology. J. Neurosci..

[29] Valanvanidis A., Vlachogianni T., Fiotakis C. (2009). 8-hydroxy-2′-deoxyguanosine (8-OHdG): A critical biomarker of oxidative stress and carcinogenesis. J. Environ. Sci. Health Part C.

[30] Lopes L., Davenport C., Torrres E., Chlebowski A., Mikami A., Raber J., Kisby G. (2023). Neuropathological Examination of Mice Chronically Exposed to Secondhand Smoke. Mil. Med..

[31] Valieva Y., Ivanova E., Fayzullin A., Kurkov A., Igrunkova A. (2022). Senescence-Associated-Galactosidase Detection in Pathology. Diagnostics.

[32] de Mera-Rodríguez J.A., Álvarez-Hernán G., Gañán Y., Martín-Partido G., Rodríguez-León J., Francisco-Morcillo J. (2021). Is Senescence-Associated β-Galactosidase a Reliable in vivo Marker of Cellular Senescence During Embryonic Development?. Front. Cell Dev. Biol..

[33] Martínez-Zamudio R.I., Dewald H.K., Vasilopoulos T., Gittens-Williams L., Fitzgerald-Bocarsly P., Herbig U. (2021). Senescence-associated β-galactosidase reveals the abundance of senescent CD8+ T cells in aging humans. Aging Cell.

[34] Gorgoulis V., Adams P.D., Alimonti A., Bennett D.C., Bischof O., Bishop C., Campisi J., Collado M., Evangelou K., Ferbeyre G. (2019). Cellular senescence: Defining a path forward. Cell.

[35] Paudel K.R., Panth N., Manandhar B., Singh S.K., Gupta G., Wich P.R., Nammi S., MacLoughlin R., Adams J., Warkiani M.E. (2022). Attenuation of Cigarette-Smoke-Induced Oxidative Stress, Senescence, and Inflammation by Berberine-Loaded Liquid Crystalline Nanoparticles: In Vitro Study in 16HBE and RAW264.7 Cells. Antioxidants.

[36] Kundu P., Stagaman K., Kasschau K., Holden S., Shulzhenko N., Sharpton T.J., Raber J. (2022). Fecal implants from AppNL-G-F and AppNL-G-F/E4 donor mice sufficient to induce behavioral phenotypes in germ-free mice. Front. Behav. Neurosci..

[37] Tominaga T., Shimada R., Okada Y., Kawmata T., Kibayashi K. (2019). Senescence-associated-β-galactosidase staining following traumatic brain injury in the mouse cerebrum. PLoS ONE.

[38] Kundu P., Torres E.R.S., Stagaman K., Kasschau K., Okhovat M., Holden S., Ward K., Nevonen B., Davis T., Saito T. (2021). Integrated analysis of behavioral, epigenetic, and gut microbiome analyses in *App^NL-GF^, App^NL-F^*, and wild type mice. Sci. Rep..

[39] Raber J., Anaya A.F., Torres E., Lee J., Boutros S., Grygoryev D., Hammer A., Kasschau K., Sharpton T., Turker M. (2020). Effects of Six Sequential Charged Particle Beams on Behavioral and Cognitive Performance in B6D2F1 Female and Male Mice. Front. Physiol..

[40] Rachmian N., Krizhanovsky V. (2023). Senescent cells in the brain and where to find them. FEBS J..

[41] Lee M.Y., Ojeda-Britez S., Ehrbar D., Samwer A., Begley T.J., Melendez J.A. (2022). Selenoproteins and the senescence-associated epitranscriptome. Exp. Biol. Med..

[42] Dong L., Wu J., Li A., Bi W., Liu T., Cao L., Liu Y., Liu A. (2016). The inhibitory mechanism of Cordyceps sinensis on cigarette smoke extract-induced senescence in human bronchial epithelial cells. Int. J. Chronic Obstr. Pulm. Dis..

[43] Torres E., Akinyeke T., Stagaman K., Duvoisin R., Meshul C.K., Sharpton T.J., Raber J. (2018). Effects of sub-chronic MPTP exposure on behavioral and cognitive performance and the microbiome of wild-type and mGlu8 knockout female and male mice. Front. Behav. Neurosci..

[44] Barrientos S.A., Tiznado V. (2016). Hippocampal CA1 Subregion as a Context Decoder. J. Neurosci..

[45] Graille M., Wild P., Sauvain J.J., Hemmendinger M., Guseva Canu I., Hopf N.B. (2020). Urinary 8-OHdG as a Biomarker for Oxidative Stress: A Systematic Literature Review and Meta-Analysis. Int. J. Mol. Sci..

[46] Gao X., Lai C.Q., Scott T., Shen J., Cai T., Ordovas J.M., Tucker K.L. (2010). Urinary 8-Hydroxy-2-deoxyguanosine and Cognitive Function in Puerto Rican Adults. Am. J. Epidemiol..

[47] Peña-Bautista C., Tirle T., López-Nogueroles M., Vento M., Baquero M., Cháfer-Pericás C. (2019). Chafer-Pericas Oxidative Damage of DNA as Early Marker of Alzheimer’s Disease. Int. J. Mol. Sci..

[48] Abou-Elela D.H., El-Edel R.H., Shalaby A.S., Fouaad M.A., Sonbol A.A. (2020). Sonbol Telomere length and 8-hydroxy-2-deoxyguanosine as markers for early prediction of Alzheimer disease. Indian J. Psychiatr..

[49] Liu Z., Liu Y., Tu X., Shen H., Qiu H., Chen H., He J. (2017). High Serum Levels of Malondialdehyde and 8-OHdG are both Associated with Early Cognitive Impairment in Patients with Acute Ischaemic Stroke. Sci. Rep..

[50] Liu Z., Cai Y., He J. (2018). High serum levels of 8-OHdG are an independent predictor of post-stroke depression in Chinese stroke survivors. Neuropsychiatr. Dis. Treat..

[51] Shandilya S., Kumar S., Jha N., Kesari K., Ruokolainen J. (2022). Interplay of gut microbiota and oxidative stress: Perspective on neurodegeneration and neuroprotection. J. Adv. Res..

[52] Zhu S., Jiang Y., Xu K., Cui M., Ye W., Zhao G., Jin L., Chen X. (2020). The progress of gut microbiome research related to brain disorders. J. Neuroinflamm..

[53] Dumitrescu L., Popescu-Olaru I., Cozma L., Tulbă D., Hinescu M.E., Ceafalan L.C., Gherghiceanu M., Popescu B.O. (2018). Oxidative Stress and the Microbiota-Gut-Brain Axis. Oxid. Med. Cell Longev..

[54] Yang J.-L., Chen W.-Y., Mukda S., Yang Y.-R., Sun S.-F., Chen S.-D. (2020). Oxidative DNA damage is concurrently repaired by base excision repair (BER) and apyrimidinic endonuclease 1 (APE1)-initiated nonhomologous end joining (NHEJ) in cortical neurons. Neuropathol. Appl. Neurobiol..

[55] Zhang Q., Yang L., Gao H., Kuang X., Xiao H., Yang C., Li M. (2023). APE1 promotes non-homologous end joining by initiating DNA double-strand break formation and decreasing ubiquitination of artemis following oxidative genotoxic stress. J. Trans. Med..

[56] Vazquez-Villasenor I., Garwood C.J., Simpson J.E., Heath P.R., Mortiboys H., Wharton S.B. (2021). Persistent DNA damage alters the neuronal transcriptome suggesting cell cycle dysregulation and altered mitochondrial function. Eur. J. Neurosci..

[57] Sah E., Krishnamurthy S., Ahmidouch M.Y., Gillispie G.J., Milligan C., Orr M.E. (2021). The Cellular Senescence Stress Response in Post-Mitotic Brain Cells: Cell Survival at the Expense of Tissue Degeneration. Life.

[58] Kumari R., Jat P. (2021). Mechanisms of Cellular Senescence: Cell Cycle Arrest and Senescence Associated Secretory Phenotype. Front. Cell Dev. Biol..

